# Physical Diagnosis in Determining the Amount of Muscular Effort Demanded in the Operation for the Extraction of Teeth

**Published:** 1848-01

**Authors:** C. O. Cone

**Affiliations:** Baltimore.


### ARTICLE VI.

*Physical Diagnosis in Determining the Amount of Muscular Effort Demanded in the Operation for the Extraction of Teeth.* By C. O. CONE, D. D. S., Baltimore.

THE extraction of teeth is an operation of so frequent an occurrence, and so generally performed without immediately dangerous consequences following, that it has been considered an unlicensed field to labor in, by all that is prompted by a disposition, till this very democratic permission finds a *painful* response, by the strong feelings of dread and reluctance, with which most patients submit to the operation.

To the reflecting dental surgeon, who appreciates the feelings of those who ask his professional assistance, and is emulated with the laudable desire to attain the highest point of professional excellence, will not look upon any thing that may tend to illustrate or explain any feature of this operation with indifference.

The pain necessarily attendant on the removal of a tooth can be greatly abridged, and the certainty of a successful issue much enhanced, by adopting such preparatory measures as the circumstances of the case would seem to demand in securing the desired end. But that there are hundreds in our own profession who never adopt any precautionary measures, or examine the indications connected with the proposed operation, or certain caution to be exercised, but rather trust to the fortunate chance of accident, is a truth that does not demand for its establishment, illustration. And it is equally true, that a large proportion of such practitoners would gladly avail themselves of the benefits of finger-board-facts in their guidance, if such existed and were tangible. To such, and the *tyro* in the profession, I need not offer any apology for the hints which I am about to propose.

The teeth are held in their respective places in the dental arch by their surrounding alveolar walls, which are accurately adapted to the size and form of the fangs they enclose, and it is to these, aided by the intimate connection existing between the periosteal membrane covering their fangs, together with the length of the latter, and their divergence from parallelism that their firmness is established, although the degree of their firmness is determined by the peculiarities of tissue and structure, or constitutional formation; and that these can be very generally determined by physical indications is unequivocally certain.

To distinguish, and correctly interpret these physical signals with unerring certainty, I am candid to confess is almost impossible, or can only be done with great difficulty, and even then by such only, who have long exercised themselves in this field of physical observation. But to determine whether a tooth demands much or little physical force to remove it from its articulation, is a question more easily determined; and it is a qualification which the dentist should possess in a very general degree; for should he attempt to extract a tooth, without applying sufficient force to the instrument operating upon the tooth, to break up its attachment in the direction corresponding to the motion given by the instrument operating on the tooth, the motion might as well not have been given, so far as the luxation of the organ is concerned in that step of the operation; although it may exhibit the demands required for the next step, which knowledge should have been possessed by the operator, even anterior to his making choice of the instrument for the extraction.

The degree of firmness with which the alveolar processes retain the teeth in their sockets, is determined in a very great measure by the density and unyielding texture of its structure, or, in other words, upon not only the quantity and quality of its mineral ingredients, but, also, upon their molicular and cellular arrangement, and component quality of its fibrous structural base, and the relative amount of water that is held in its union, together with the thickness of their internal and external plates, and the volume of their transverse uniting laminæ. All of which is dependent on, and regulated by the component parts of the blood. If the red particles of the blood predominates, the osseous parts will be firm and context in structure, and its formation resembling more the compact, than the *areolar texture.*

The periostial tissue lining the cavities in the alveolus for the reception of the fangs of the teeth, and which changes its character in some degree, becoming more adhesive and dense as it ascends about the necks of the last named organs, making their union with the surrounding parts more secure, is dependant also for its firmness and tenacity, together with the surrounding soft tissues upon the condition of the blood, which is determined by the predominance of gelatine over the albuminous particles of this fluid.

Having thus, very briefly, considered the causes that determine the various conditions and degree of firmness of the parts surrounding the teeth, and before proceeding to describe the physical peculiarities marking the various degrees of firmness, of the osseous and fibrous structure in individuals, I would observe, that in all cases, whether the support to the teeth be great or small, their articulation is more firmly established at that period marking the meridian of life, as the chemical composition of the osseous parts, as well as the tenacity of the fibrous tissues varies considerably in the various stages of life, and we may also add, under different circumstances of health and disease. In childhood and youth the relative proportions of animal matter is at its maximum, rendering the bony structure more easily compressed, and the fibrous tissue easily torn, while in advanced age, from the great preponderance of phosphate and carbonate of lime, the osseous tissue is friable and easily fractured, and the fibrous structure rendered brittle.

We find in those individuals who are near a perfect state of health, resulting from good constitutional organization, and where the sanguinous temperament predominates, have all of their organs well developed, the alveolar arch describing regular elliptical circles, and presenting a great degree of strength, by the thickness of their plates, and the perpendicular height with which they rise from their base. It will also be observed, as the lancet is introduced for the separation of the gum from the neck of the tooth, that the alveolar plates are very resisting and compact, and their close adaptation to the fangs of teeth will not permit the instrument to detect the line of separation, but which would appear like one continuous bony structure. The fibrous structure presents also great density of formation. Such, usually, have accompanying the preceding characteristics, teeth regularly arranged, the cutting edges of the superior anterior teeth meeting or striking slightly oblique or vertical; the molars but faintly marked with cusps on their grinding surfaces, the crowns of all classes short and thick; the anterior teeth presenting almost a rounded appearance; the length of all classes show great uniformity, and their necks much narrower than at their cutting edges, their color varying from a cream white to a pearly cast, tinged with yellow, the earthy salts predominating, rendering them hard and not easily acted upon by corrosive agents. The external plate of their alveolus presenting one continuous line, unbroken by prominences, marking the size and directions of the fangs of the teeth. With individuals possessing such physical indications, after the system has acquired its full strength, and has not been enervated by constitutional or local disease, or old age, demand the *greatest* amount of muscular effort, and cautionary measures to be adopted for the extraction of their teeth.

The next class of teeth that demand great physical exertion for their luxation, is found in individuals whose temperaments are sanguinous, although not so plainly marked as in individuals possessing teeth bearing characteristics like those just described. The teeth are long and well formed, and somewhat rounded; the cuspidati usually pointed, although frequently worn to an obtuse point; the cutting edges of the superior lateral incisores are rounded. Such teeth are usually crowded in their arrangement in their alveolar arches, which are usually prominent and thin, and not, unfrequently, from this cause, the anterior superior teeth made to strike, perpendicularly, on the inferior teeth, or within the arch of the same. The color of such teeth may be either a light cream, or a muddy white; and when the latter exist, their structure is such as to render their crowns brittle. Organs of this description are generally found with individuals having thin but prominent countenances, and energetic features.

Another class of teeth having a relationship not greatly distant from the last described, in their firmness of articulation, are distinguished by their light complexion, which is often tinged with a feint, hardly perceptible blue, at their cutting edges; the incisores long, flat and thin; the superior incisores greatly overlapping the inferior. Their alveolar borders are thin, and closely adapted to the necks of the teeth. The gums are of a light pink complexion, firm in their consistency, and their apices plainly and distinctly marked.

There is another class of teeth that often demand considerable physical exertion in their removal, and their distinguishing marks are a regular arrangement, although, sometimes, but rarely ever crowded; they are, in shape, short and narrow, frequently of a cream complexion; the cuspidati rounded and pointed; the alveolar border marked by prominences corresponding to the fangs of the teeth, and that portion surrounding the anterior superior teeth, and are usually prominent, and exposed to view when the lip is raised in the act of smiling. A peculiar characteristic marks this class of teeth, is shown by a space intervening between the crowns of the anterior, and, frequently, between the posterior teeth, increasing the thickness and consequent firm support of the transverse bony laminæ, connecting the external and internal alveolar border. The fangs of the teeth, now under consideration, frequently assume an irregular direction, and are disproportionately long. The gums, as in the last class, when not diseased, are of a firm texture, and their union with the necks of the teeth well marked, and their color a faint pink.

A class of teeth possessing the following characteristics will be recognised by the observant dentist, as demanding much less muscular effort for their removal than those already described, but which are liable to deceive all but an accurate observer, by the length of their crown, &c. The anterior teeth are usually broad but not thin ; the superior incisores overlap the inferior, the cusps on the molars are prominent, and their grinding surfaces marked by irregular fissures. The lingular surface of the incisores, also, frequently have a fissure running in the direction of the long axes of the crown. Such teeth are usually found with individuals of full ordinary stature, with prominent frontal sinusses, and the *zygomatic* process of the malar bone forming an eminence unrounded by muscle, showing that the fibrous tissue is deficient in tone. Such teeth are usually of a fair complexion, and easily acted on by corrosive agents from deficiency of earthy salts.

Teeth resembling the organs last described, in shape and arrangement, but of a darker complexion, with their alveolar plates thinner, and the gums not of as loose a texture, but firm, and their union with the teeth well marked, and the osseous eminences of the body rendered symmetrical and rounded by muscular fibre, will present great resisting force in their extraction.

The next class of teeth that I would describe, are sometimes, of an opaque chalky white, and at other times of a yellow muddy white in complexion, their surfaces irregular and rough, the palatine portion of the superior molars are frequently marked by deep indentations and high eminences on the grinding surface of all the molar teeth. Their arrangement is regular, perhaps their crowns stand out a little more obliquely than usual. Their alveolar processes describe a broader circle, and are thicker, but not dense, being very cellular. The gums are usually pale, but sometimes red and flabby. The crowns of the teeth are much larger than those of teeth of ordinary size, and it would seem that they were gained at the sacrifice of the quality of materials entering into their composition. Those teeth are extracted with a small amount of physical force, and usually possessed by individuals having prominent foreheads and thick upper lips, pale pasty complexions, with affectionate and susceptible dispositions, and often animated countenances.

The next and last class of teeth that I shall describe, and which are not held firmly in their articulation, or demand great, or even considerable effort for their removal, have a beautiful azure or pearly complexion, which are sometimes regularly arranged in their arches, but more frequently irregular to a greater or less extent, from a faulty organic development of the alveolar arches, which although thin, are very cellular in their structure. The gums surrounding such teeth are usually pale, and their edges about the necks of the teeth plainly marked when they are in a healthy state, but which are subject to vascular injection, and peculiarly susceptible to morbid impressions, and when in an inflamed state, even if but slight unhealthy impressions be made, their functions are greatly changed, and the secretions of the mouth becomes much altered, acidulated and viscid. Teeth having the above marks or characteristics are usually met with in *caloric* females, and males possessing weak constitutions, with flabby muscles, pale countenances, and the white tissues greatly predominating.

A great number of subdivisions of these classes of teeth take place, and frequently one class is complicated with, or have marks which are attached to another class, but, in all such cases, sufficient diagnostic symptoms are left to direct the observant and reflective dentist to a correct conclusion.

It must be remembered that in all cases, where there is a full and unbroken dental arch, that some teeth of the same class will demand a much greater amount of muscular effort for their removal than others. When dentition is complete, and the crown of the teeth take up an irregular position in the arch, and are so situated that one or more are sustained not only by their alveolar articulations, but by the crowns of the neighboring teeth; we have in such, a firmness of articulation that is a stranger to that class of dentition. Such may also occur in any tooth or a number of teeth where their fangs are distorted and abnormal in their conformation. An unusual firmness of one or more teeth frequently exists in mouths of children when dentition has not been completed, and more frequently found in the first inferior permanent molares, although sometimes in the same class of teeth in the superior jaw. When the second molar in children is about to pass through the gum, or has just cut the same, and from a want of length in the alveolar arch lies slightly oblique, its crown pressing against the posterior approximal surface of the crown of the first molar. In such cases, a good deal of physical force is necessary to be applied to the instrument to remove the tooth above mentioned, although, as I have before intimated, the surrounding parts of the tooth at the age above specified, easily yield, and comparatively demand but a small amount of physical exertion for their removal. We also find a demand for great muscular effort in extracting the first, or second molares of either jaw or side, when their articulation is complete, and the approximal surfaces of their crowns hold each other in position by contact. Such is peculiarly the case in the inferior jaw, when the alveolar border has not lengthened sufficiently, and the *dens sapientiæ* take up a crowded position. We also find that the fangs of the first molar of either jaw, and sometimes the second molar, diverge from the neck of the tooth or its bifurcation, until their extremities describe a circle twice the diameter of the socket at its orifice, and sometimes so tortuous as to encompass or grasp a large piece of the alveolus. These peculiar conformations are more frequently met with in the first permanent molares of the first four classes described, the superior molares more frequently grasping the alveolus, and the fangs of the inferior diverging anteriorly and posteriorly, securing their articulation similar to mechanical dove-tailing.

A tooth or a set of teeth showing great firmness of articulation, may be extracted with little physical force, when its surrounding structure has changed its original character from local or general disease, or when the secreting vessels deposit carbonate and phosphate of lime in too great proportions, as in old age, and render their alveolar walls friable, and the tissues easily broken, or when the osseous and fibrous structure loses its original firmness from an enervated system, and the mineral deposit is deficient, and, at the same time, their molicular arrangement, with its fibrous base is faulty; being dependant on an unhealthy or improperly proportioned state of the circulating fluid, and thus is rendered lax and easily compressible, not possessing its original elasticity and unyielding structure.

---

### ARTICLE VII.

### *Letter from* Dr. S. C. Harbert.

